# *In vitro* and *in vivo* synergistic effect of chrysin in combination with colistin against *Acinetobacter baumannii*

**DOI:** 10.3389/fmicb.2022.961498

**Published:** 2022-10-28

**Authors:** Yining Zhao, Yan Liu, Luozhu Feng, Mengxin Xu, Hong Wen, Zhuocheng Yao, Shiyi Shi, Qing Wu, Cui Zhou, Jianming Cao, Tieli Zhou

**Affiliations:** ^1^Key Laboratory of Clinical Laboratory Diagnosis and Translational Research of Zhejiang Province, Department of Clinical Laboratory, The First Affiliated Hospital of Wenzhou Medical University, Wenzhou, China; ^2^Department of Medical Laboratory Science, School of Laboratory Medicine and Life Science, Wenzhou Medical University, Wenzhou, China

**Keywords:** colistin resistance, *Acinetobacter baumannii*, synergistic effect, chrysin, colistin

## Abstract

*Acinetobacter baumannii* is an opportunistic pathogen that is primarily associated with nosocomial infections. With the rise in cases of acquired drug resistance, *A. baumannii* is gaining resistance to conventional antimicrobial drugs and even to the last line of antibiotics, such as colistin. Hence, the application of the synergistic combination of an antibiotic and a non-antibacterial agent is being contemplated as a new alternative therapeutic approach. Chrysin is a component of honey with anti-inflammatory and antioxidant properties. In this study, we evaluated the antibacterial activity of chrysin in combination with colistin against *A. baumannii* both *in vitro* and *in vivo*, as well as the cytotoxicity of chrysin with or without colistin. Our results revealed that chrysin and colistin exerted synergistic effects against *A. baumannii* by damaging the extracellular membrane and modifying the bacterial membrane potential. The chrysin/colistin combination group demonstrated an inhibitory effect on biofilm formation. In conclusion, it is expected that the synergy between these drugs can allow the use of a lower concentration of colistin for the treatment of *A. baumannii* infections, thereby reducing dose-dependent side effects. Thus, a combination therapy of chrysin/colistin may provide a new therapeutic option for controlling *A. baumannii* infections.

## Introduction

*Acinetobacter baumannii*, a Gram-negative bacterium, is the most problematic community- and nosocomial-associated opportunistic pathogen (Sarshar et al., 2021). It has also been listed in the ESKAPE group along with *Enterococcus faecium, Staphylococcus aureus, Klebsiella pneumoniae, Pseudomonas aeruginosa*, and *Enterobacter* species (De Oliveira et al., 2020). *A. baumannii* has continued to pose a serious threat to public health over the past few decades due to its ability to easily develop resistance to most antibiotics (Magiorakos et al., 2012).

Colistin is an “old” class antibiotic as well as the last-line therapeutic option to treat infectious diseases caused by multidrug-resistant (MDR) bacteria (Nang et al., 2021). Unfortunately, with the widespread use of colistin, resistant strains have been reported worldwide, especially in Asia and Europe (Cai et al., 2012). Therefore, it is urgent to restore the bactericidal effect of colistin. A large body of literature supports that a combination therapy can reverse colistin-resistant (COL-R) strains. For example, Le Menestrel et al. (2021) reported the synergistic effect of colistin in combination with fosfomycin in a mouse infection model, and daptomycin exhibited a synergistic action against MDR *A. baumannii* (Galani et al., 2014). Interestingly, some non-antibacterial agents could enhance the therapeutic efficacy of colistin, such as melatonin (Liu et al., 2020), curcumin (Kaur et al., 2018), and anthelmintic oxyclozanide (Ayerbe-Algaba et al., 2019).

Chrysin belongs to flavonoids and is ubiquitous in plants extracted from fruits, vegetables, and honey (Mani and Natesan, 2018). Some past studies discovered that chrysin possesses anticancer (Sherif et al., 2020), antioxidative, and anti-inflammatory (Choi et al., 2017) properties. Furthermore, chrysin has been demonstrated to protect the liver and the kidney from various biochemical and molecular materials, especially colistin (Pingili et al., 2019), which is prone to cause acute kidney injury during treatment.

This is the first report of a synergistic interaction between chrysin and colistin against *A. baumannii* with varying levels of colistin sensitivity. In this study, we determined synergistic antimicrobial activity both *in vitro* and *in vivo*, and mechanistic studies revealed that chrysin can alter bacterial cell membrane permeability. In summary, the dosage of colistin decreased drastically as a result of the effect of chrysin on *A. baumannii*.

## Materials and methods

### Bacterial strains and chemicals

A total of five non-duplicated COL-R and two colistin-sensitive (COL-S) *A. baumannii* isolates were recovered from the First Affiliated Hospital of Wenzhou Medical University in China. All these strains were identified by matrix-assisted laser desorption/ionization time-of-flight mass spectrometry (MALDI-TOF/MS; bioMérieux, Lyons, France). ATCC 19606 served as a quality control strain in this study. These strains were conserved in Luria Bertani (LB) broth medium containing 30% glycerol at −80°C until use. All bacterial strains were routinely grown on Luria–Bertani agar plates or LB broth at 37°C before conducting these experiments. Colistin was purchased from Wenzhou Kangtai Biological Technology Co., Ltd. (Zhejiang, China), and chrysin was purchased from MedChemExpress (MCE) Co., Ltd. (NJ, USA). Colistin and chrysin were dissolved in double-distilled water (ddH_2_O) and diluted with the culture medium to the desired concentrations for *in vitro* studies. Colistin and chrysin were dissolved in ddH_2_O and diluted with normal saline to the required concentration for *in vivo* studies.

### Antibiotic susceptibility and checkerboard assay

Minimum inhibitory concentrations (MICs) of colistin and the MIC of chrysin against clinical COL-R and COL-S *A. baumannii* were determined by broth microdilution assay with cation-adjusted Mueller–Hinton broth (CAMHB) in accordance with the Clinical and Laboratory Standards Institute (CLSI, 2020) recommendations. As previously reported, the checkerboard assay was performed to explore the synergistic effect of colistin in combination with chrysin. Briefly, 100 μl of the initial inoculum of 1.5 × 10^6^ colony forming unit (CFU)/ml for each strain was added to a 96-well microtiter plate, where each well contained 100 μl of colistin (0–32 μg/ml), chrysin (0–128 μg/ml), or colistin plus chrysin (the final concentration is the same as the one that is previously described) and were then cultured for 16–18 h at 37°C (Al Atya et al., 2016).

The fractional inhibitory concentration index (FICI) was applied to evaluate the synergistic effect of a two-drug combination (Lories et al., 2020). The formula was used to calculate the results: FICI = (MIC_A_ in combination/MIC_A_ alone) + (MIC_B_ in combination/MIC_B_ alone). The interaction of a drug combination was classified according to the following criteria: (1) FICI ≤ 0.5, synergistic effect; (2) 0.5 < FICI ≤ 1, additive effect; (3) 1 < FICI < 4, no interaction; and (4) FICI ≥4.0, antagonism.

### Time-dependent killing

The tested strains were subjected to a time-kill assay using colistin, chrysin, and a combination of colistin with chrysin. Bacterial cultures without drugs were performed in parallel and used as controls. Then, 200 μl of 1.5 × 10^8^ CFU/ml of bacteria were added into tubes containing 20 ml of CAMHB, with or without an antibiotic. These tubes were incubated at 37°C and kept shaking for 24 h at 200 revolutions per minute (rpm). At each time point (i.e., 2, 4, 6, 12, and 24 h), 200 μl of the bacterial suspension was serially diluted 10-fold and then transferred into LB agar plates to enumerate the number of bacteria. These plates were incubated statically at 37°C for 18 h. The synergy effect was defined as a >2 log_10_ decrease in CFU/ml after 24 h of the combination treatment relative to that after treatment with colistin or chrysin individually (Percin et al., 2014).

### Biofilm inhibition assay

The synergistic effect of chrysin and colistin on a biofilm was determined using a 96-well polystyrene plate. The log-phase bacterial suspension of 1.5 × 10^6^ CFU/ml was cultured in wells containing chrysin (2, 4, or 8 μg/ml) and colistin (0.25 μg/ml) either alone or in combination. LB broth containing 1.5 × 10^6^ CFU/ml bacteria served as the negative control. The inoculated plates were incubated at 37°C for 24 h. Afterward, planktonic cells were removed and washed two times with 200 μl of 1 × phosphate-buffered saline (PBS) (Sigma-Aldrich, Milan, Italy). The plate was dried at room temperature to fix the attached biofilm. Subsequently, 150 μl of 1% crystal violet was added to the wells to stain the total biomass of a biofilm for 15 min. The cells were washed with ddH_2_O. Then, an equal amount of 95% ethanol was added to each well to dissolve the crystal violet. Finally, a 96-well plate was placed in a microplate reader to determine the absorbance of the sample at 595 nm (OD_595_) (Ulrey et al., 2014).

### Cytotoxicity assay

*In vitro* cytotoxicity assays were performed with RAW264.7 (ATCC, Manassas, VA) cells. RAW264.7, a type of monocyte-macrophage from mice, was cultured until they reached confluency in Dulbecco's Modified Eagle's Medium (DMEM) supplemented with 10% heat-inactivated fetal bovine serum (FBS). The confluent cells were dispersed in trypsin. Then, 100 μl of the cell suspension containing 1 × 10^5^ cells was added to a 96-well plate. After culturing for 24 h, 10 μl of different final concentrations of chrysin (2, 4, 8, and 16 μg/ml) and 0.25 μg/ml of colistin were added to the medium and cultured for another 12 h. The complete media containing the cells only was used as the positive control. After incubation, 10 μl of the cell counting kit-8 (CCK-8) (Dojindo Laboratories, Japan) was added to each well in a 96-well plate and incubated at room temperature for 1 h. The absorbance was measured at 450 nm using a microplate reader (Zhang et al., 2019a).

### Determination of the synergistic effect *in vivo*

The infection model of *Galleria mellonella* larvae: *G. mellonella* larvae weighing between 250 and 350 mg were selected for the test and randomly assigned into five groups, including the positive-control group, the single-treatment groups, the combination-treatment group, and the PBS alone treatment group (not injected with bacterial suspension). Each group contained 10 larvae. An overnight culture of the *A. baumannii* strain BM2622 was washed with PBS, and the bacterial load was adjusted to 1 × 10^7^ CFU/ml with PBS. The PBS-treated group served as the control group. Then, a microsyringe was used to inject 10 μl of the bacterial suspension into the right posterior gastropod of the *G. mellonella* larvae. After infection by bacteria for 2 h, these larvae were injected with 10 μl of the therapeutic drug into the left posterior gastropod, either in the single-drug group (chrysin 4 μg/ml × 7; colistin 0.25 μg/ml × 7) or in the combined-treatment group (chrysin 4 μg/ml × 7 + colistin 0.25 μg/ml × 7). Meanwhile, the positive-control group was only injected with 10 μl of PBS. These larvae were placed in the dark at 37°C, and the survival of *G. mellonella* larvae was recorded after 24, 48, 72, 96, 120, 144, and 168 h. All experiments were conducted in triplicate, and the survival rate was taken as the average percentage of the three experiments. When these larvae failed to respond to physical stimuli, they were considered to be dead. The results were assessed by the Kaplan–Meier analysis and the log-rank test in terms of the rate and extent of mortality in *G. mellonella* (Fleitas Martinez et al., 2019).

Neutropenic mouse thigh infection model: We further explored the synergistic effect of colistin and chrysin against *A. baumannii* in a neutropenic mouse thigh infection model with some minor modifications (Sabet et al., 2019, 2020). We selected 5–6-week-old specific pathogen-free (SPF) female ICR mice for this experiment, and the clinically isolated COL-R *A. baumannii* BM2622 was selected. Before the start of this infection, mice were made neutropenic *via* intraperitoneal administration of 150 mg/kg cyclophosphamide (Yuanye Biotechnology Co., Ltd., Shanghai, China) at 4 days and 1 day. Then, they were infected *via* an intramuscular injection of 0.1 ml of the inoculum (10^6^ CFU/ml) into the thigh muscles. Dosages of 7.5 mg/kg/24 h colistin and 50 mg/kg/24 h chrysin were administered either as monotherapy or as a combination therapy, and all mice were injected with the prescribed medicine one time per day. After the final treatment, all mice were euthanized within 24 h, and the thighs were homogenized followed by plating on Mueller–Hinton agar plates. After incubation for 24 h at 37°C, the colonies were counted as the bacterial burden. Differences in the colony counts were analyzed using a two-tailed *t*-test (GraphPad Prism, version 6). A *p*-value < 0.05 was considered to be statistically significant.

### Membrane permeability and the potential of bacteria

To explore the mechanisms of synergy between chrysin and colistin, propidium iodide (PI) staining and membrane potential assay were conducted with some minor modifications (Uppu et al., 2015; Zhang et al., 2019b).

Cell membrane permeability: PI staining was performed to measure cell membrane permeability. PI (50 μg/ml) was added to the bacterial suspension after bacteria were treated with 0.125 μg/ml of colistin and different concentrations of chrysin (2, 4, and 8 μg/ml) with or without colistin for 2 h. Next, the fluorescence intensity was measured on a microplate reader. The excitation wavelength and the emission wavelength were set to 535 and 615 nm, respectively.

Membrane potential: 3,3′-diethyloxacarbocyanine iodide [DiOC2(3)] (MaoKang Biotechnology, Inc., Shanghai, China) was used to assess the membrane potential, as previously studied with some minor modifications. Since DiOC2(3) is a fluorescent membrane potential probe that originally emits green fluorescence, the fluorescence of DiOC2(3) turned red when the membrane voltage increased. The fluorescence of DiOC2(3) was tested using a 96-well-black flat-bottom plate. After the log-phase growth, bacteria were processed similar to those in the PI staining assay, a final concentration of 30 μm of the probe was added and incubated at room temperature for 30 min, and the group treated with CCCP served as the positive control in the experiment. Cell membrane potential was measured using a multi-plate reader (BioTek) with an excitation light of 486 nm and an emission light of 620 nm. All assays were conducted three times.

### Microscopic examination of a biofilm

The synergistic effects of chrysin and colistin on biofilm cells were examined using Confocal Laser Scanning Microscopy (CLSM), as demonstrated in a previous study with some modifications (Cambronel et al., 2020). The log-phase bacterial suspension of 1.5 × 10^6^ CFU/ml of BM2622 was added into a 6-well microplate, and each well contained 2 ml of the LB broth medium with 8 μg/ml of chrysin and 0.25 μg/ml of colistin alone or in combination. The plate was incubated at 37°C for 24 h. Next, the microplate was washed three times with ddH_2_O and stained with a 5-μm SYTO 9 green fluorescent nucleic acid dye (Invitrogen, Thermo Fisher Scientific) for 15 min. The plate was washed two times. Microscopic observation of the biofilm was performed using a Nikon A1 (Nikon, Japan) with a 20 × oil immersion objective. The viable bacteria were enumerated by SYTO 9 green fluorescence. The fluorescent dye was excited at 488 nm, and fluorescence emission was detected at 500–550 nm.

### Quantitative real-time polymerase chain reaction analysis

The total ribonucleic acid (RNA) from *A. baumannii* was isolated using the Trizol method, after the test strain was treated either in combination or alone with 4 μg/ml of chrysin and 0.25 μg/ml of colistin, and the extracted RNA was reversed into complementary DNA (cDNA) using a high-capacity cDNA reverse transcription kit (Applied Biosystems, USA) in accordance with the manufacturer's instructions. Quantitative polymerase chain reaction (qPCR) analysis was performed on real-time PCR for biofilm-associated virulence genes, such as *bfmR, csuA/B, ompA, pgaC, katE, bap*, and *rplB*, using PCR mixtures in predefined ratios (SYBR Green kit, Applied Biosystems, USA). Primers for the candidate genes were listed in Supplementary Table S1. *rplB* was used as a housekeeping gene. Fold changes in the gene expression were calculated using the 2^(−ΔΔCt)^ method (Sato et al., 2018; Selvaraj et al., 2020). The visualization of the results of qPCR was performed using the “seaborn (version 0.11.2)” Python package.

### Statistical analysis

Data were expressed as mean ± standard deviation (SD) of at least three independent trials. The significance was determined by using the two-sample *t*-test and the log-rank test and indicated as ^*^p < 0.05, ^**^p < 0.01, and ^***^p < 0.001. Statistical analyses were performed using Graph Pad Prism 8.0 statistical software.

## Results

### Antibiotic susceptibility testing and synergistic effect assay

Standard broth dilution assays were initially applied to determine the potency of chrysin as an antibiotic assistant against *A. baumannii*. These results revealed that MICs of chrysin were >128 μg/ml for all tested strains.

To explore the interaction of chrysin and colistin, the checkerboard assay was performed. The results listed in Table 1 indicated that chrysin could significantly decrease the MIC value of colistin against *A. baumannii* strains, which were even much lower than the susceptibility breakpoint of 2 μg/ml. The FICI values of chrysin and colistin are presented in Table 1. The sensitivity of *A. baumannii* to colistin could be enhanced in combination with chrysin.

**Table 1:** Fractional inhibitory concentration index (FICI) for colistin/chrysin combinations against *Acinetobacter baumannii*.

**Strains**	**Monotherapy (**μ**g/ml)**		**Combination (**μ**g/ml)**		**FICI**	**Interpretation**
	**Colistin**	**Chrysin**	**Colistin**	**Chrysin**		
ATCC 19606	0.125	>128	0.031	1	0.256	Synergistic
BM 1539-S	1	>128	0.125	2	0.141	Synergistic
BM 1579-S	1	>128	0.125	8	0.188	Synergistic
BM 2349	**8**	>128	0.125	8	0.078	Synergistic
BM 2412	**8**	>128	0.125	4	0.078	Synergistic
BM 2431	**16**	>128	0.125	8	0.047	Synergistic
BM 2370	**8**	>128	0.125	8	0.070	Synergistic
BM 2622	**8**	>128	0.125	8	0.078	Synergistic

Values in bold indicate resistance.

### Synergistic activity in time-killing assays

**Figure 1:**
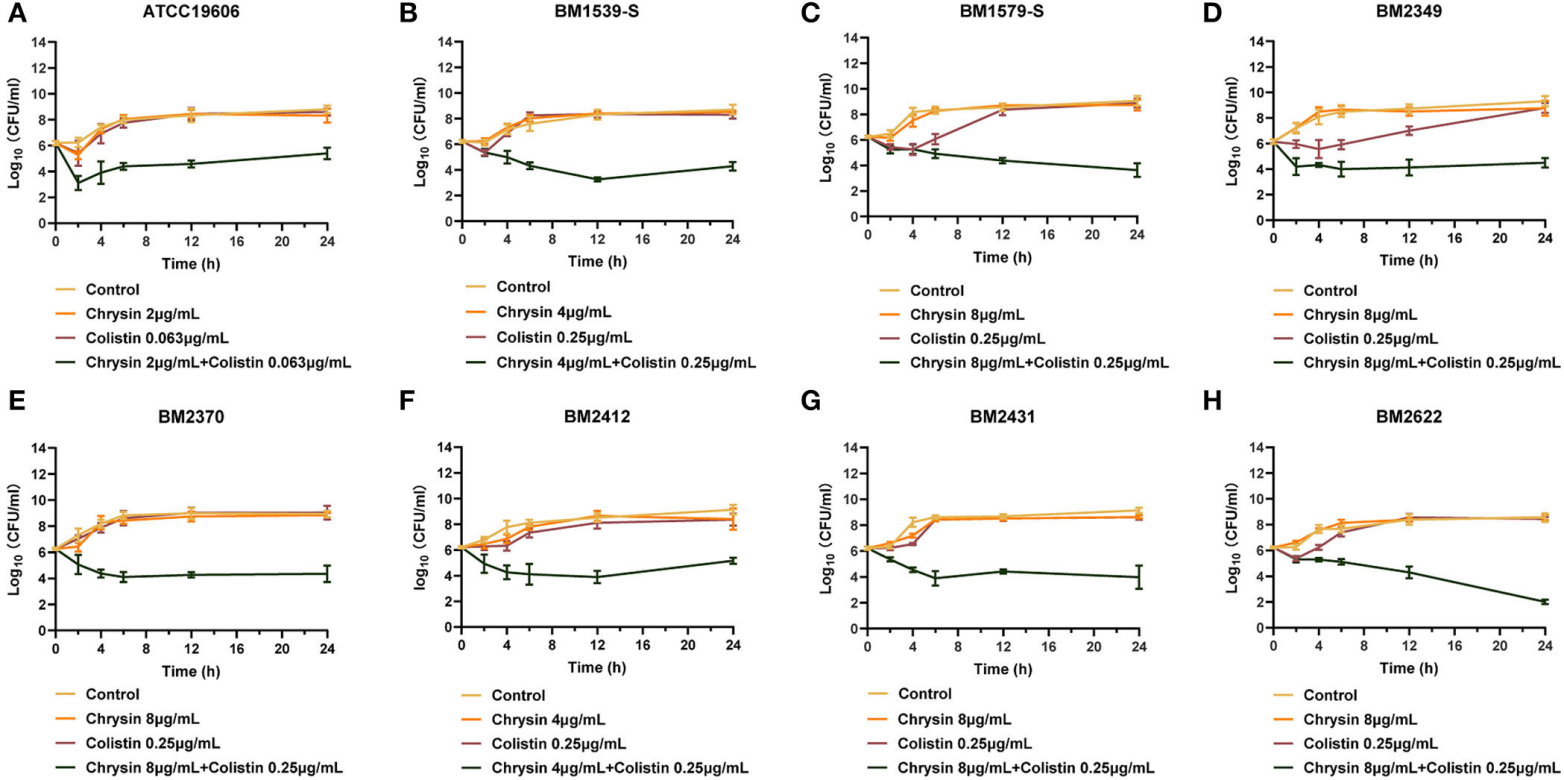
Time-killing curves of colistin and chrysin alone or in combination against *Acinetobacter baumannii*. **(A)** ATCC 19606, **(B)** BM1539-S, **(C)** BM1579-S, **(D)** BM2349, **(E)** BM2370, **(F)** BM2412, **(G)** BM2431, and **(H)** BM2622.

To further explore the combination activity between chrysin and colistin, all the tested strains were selected for time-killing assays. The concentration of chrysin and colistin was selected based on the results of the checkerboard assay, of which colistin was 0.25 μg/ml and chrysin was 4 μg/ml (for BM1539-S and BM2412) and 8 μg/ml (for BM1579-S, BM2349, BM2370, BM2431, and BM2622), and 2 μg/ml of chrysin along with 0.125 μg/ml colistin was used for ATCC19606. As shown in Figure 1, the growth of the tested strains could not be inhibited by either colistin or chrysin within 12 h. However, the combination of chrysin and colistin significantly decreased the bacterial load, which was approximately lower >3 log_10_ than the other treated groups in 24 h. In conclusion, chrysin indeed increased the activity of colistin against *A. baumannii*.

### Effect of chrysin and colistin on the inhibition of biofilm formation

**Figure 2:**
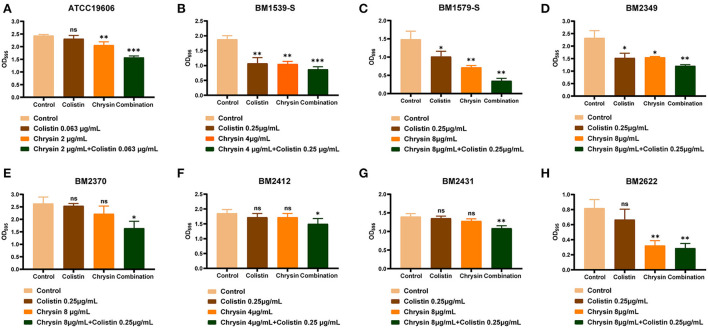
Biofilm formation-inhibition effects of colistin combined with chrysin on *A. baumannii*. **(A)** ATCC 19606, **(B)** BM1539-S, **(C)** BM1579-S, **(D)** BM2349, **(E)** BM2370, **(F)** BM2412, **(G)** BM2431, and **(H)** BM2622. *p* < 0.05 (noted with*), *p* < 0.01 (noted with**), and *p* < 0.001 (noted with***). Data are expressed as the mean ± standard deviation (SD) of three replicates.

All the tested strains could form a biofilm; hence, we performed biofilm inhibition assays to evaluate the synergistic effect of chrysin and colistin on the biofilm formed by COL-R or COL-S strains. In accordance with the aforementioned synergistic effect, we presumed that chrysin might exhibit synergy with colistin on the inhibition of bacterial biofilm formation. As shown in Figure 2, the combination of chrysin and colistin significantly inhibited the amount of biofilm formed by *A. baumannii*. This observation can be attributed to the fact that chrysin can inhibit the biofilm to an extent. However, chrysin exhibited no effect on the formation of BM2370, BM2412, and BM2431 biofilms, which warrants further exploration of the underlying mechanism.

### Safety evaluation of chrysin

**Figure 3:**
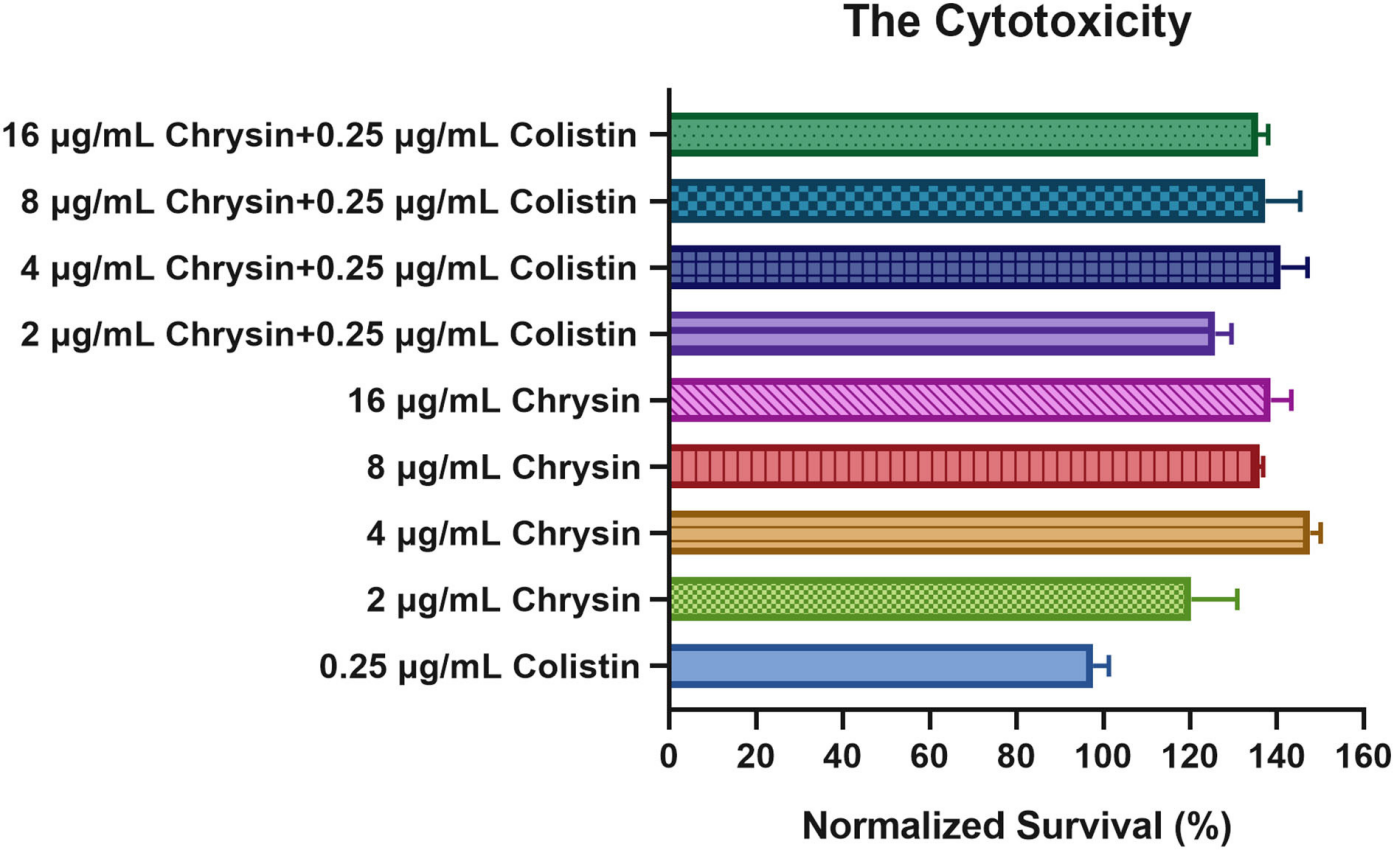
The cytotoxic effect of chrysin alone and in combination with different concentrations against the RAW 264.7 murine macrophage cell line. Data are expressed as the mean ± SD of three replicates.

Considering that chrysin has not been approved by the Food and Drug Administration (FDA) for clinical application, we selected RAW264.7 cells to test the safety of chrysin and the combination treatment. These results indicated that 2–16 μg/ml of chrysin in combination with or without 0.25 μg/ml of colistin demonstrated no toxicity to RAW264.7 cells (Figure 3). Furthermore, the normalized survival rate of the treatment group was >100%, indicating that chrysin could stimulate the macrophage. In summary, chrysin exhibited no cytotoxicity.

### *In vivo* synergy of chrysin and colistin

**Figure 4:**
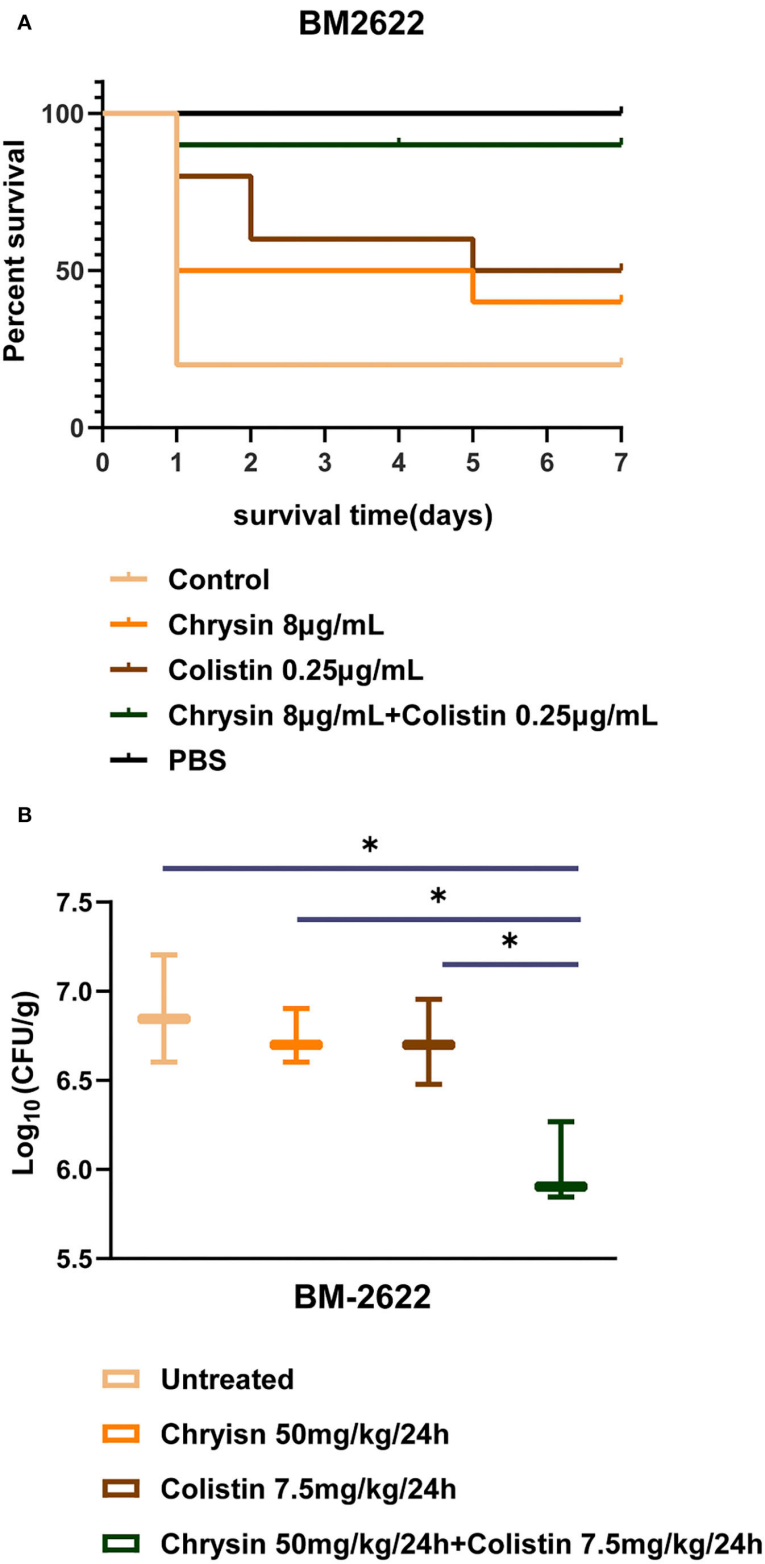
*In vivo* experiments of chrysin and colistin. **(A)** The survival rate of *Galleria mellonella* after 7 days of monotherapy or combination therapy against colistin-resistant (COL-R) *A. baumannii* BM2622. **(B)** *A. baumannii* BM2622 burden in the mouse thigh model. *p* <0.05 (noted with*), data are expressed as the mean ± SD of three replicates.

We investigated the *in vivo* efficacy of colistin in combination with chrysin on the time to death for wax moth larvae infected by BM2622. As shown in Figure 4A, the survival rate of larvae injected with the bacterial suspension decreased to 20% on day 1 (8 out of 10 dead larvae). The combination of chrysin and colistin significantly increased the number of live larvae such that 90% of the larvae in the combination group were kept alive until the last day of the experiment. The survival rate of animals was 40 and 50% in the chrysin or colistin mono-treated group within 7 days, respectively. All larvae injected with PBS alone were kept alive until the last day of the experiment. This finding indicated that the injection of chrysin in combination with colistin was the most successful at prolonging larval survival.

The results of the neutropenic mouse thigh infection model indicated that 50 mg/kg of chrysin and 7.5 mg/kg of colistin did not inhibit the growth of COL-R *A. baumannii* BM2622 in 24 h after injection. In addition, colistin in combination with chrysin exhibited a higher efficacy than monotherapy (Figure 4B). These *in vivo* experiments revealed that combined treatment with colistin and chrysin showed a significant synergistic effect against COL-R *A. baumannii*.

### Mechanism of synergistic activity against *A. baumannii*

**Figure 5:**
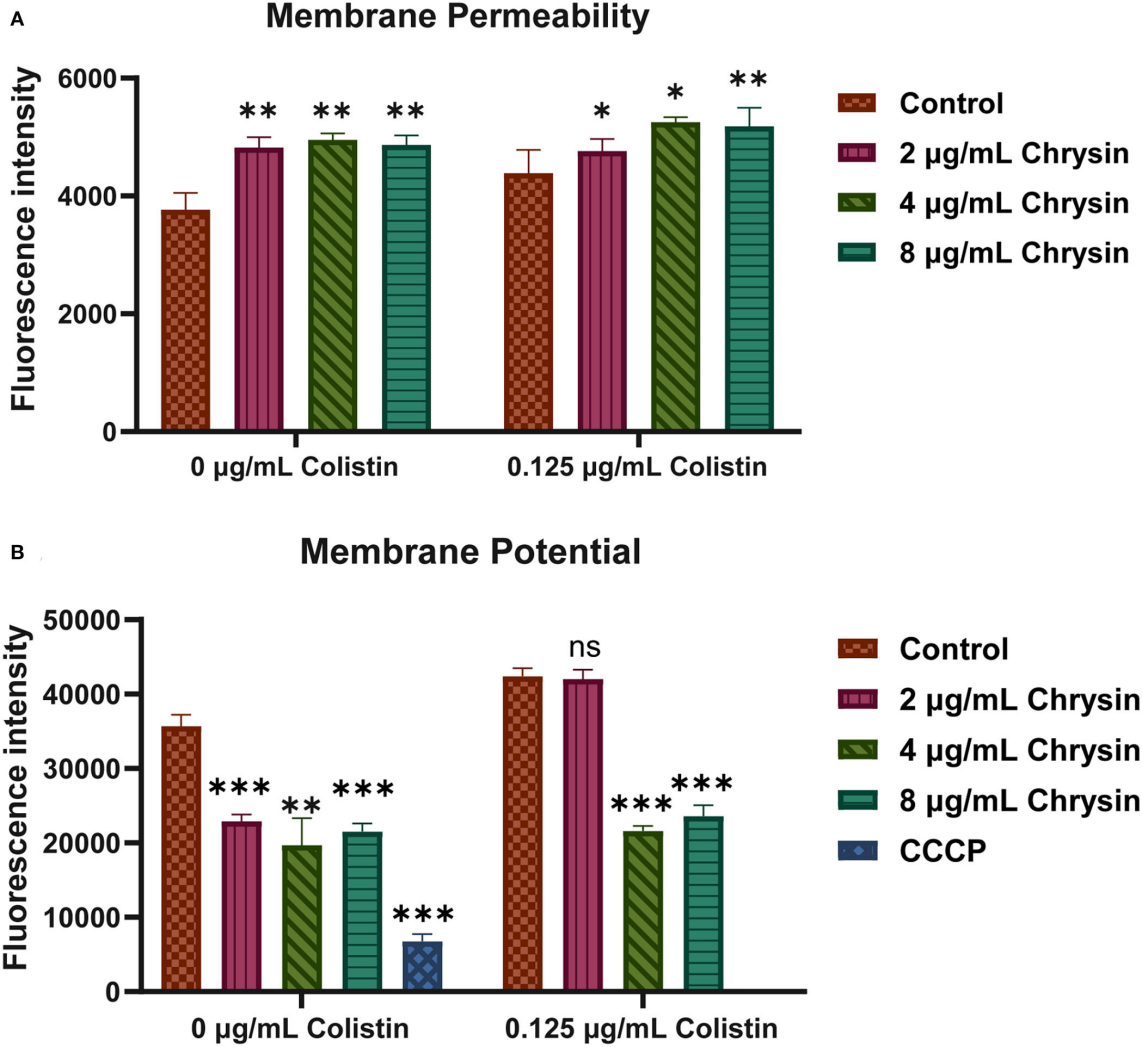
The mechanism of synergystic activity of chrysin and colistin. **(A)** The membrane permeability of *A. baumannii* BM2622 treated with chrysin and colistin or monotherapy. **(B)** The potential of the cell membrane of *A. baumannii* BM2622 treated with chrysin and colistin or monotherapy. *p* < 0.05 (noted with*), *p* < 0.01 (noted with**), and *p* < 0.001 (noted with***), data are expressed as the mean ± SD of three replicates.

We further explored membrane permeability and the potential of the tested strains. PI was performed to determine the cell membrane integrity. As shown in Figure 5A, fluorescence intensities presented an increment in the treatment group, implying that both colistin and chrysin altered the membrane permeability of *A. baumannii* and that chrysin enhanced the membrane-destructive activity of colistin.

3,3′-diethyloxacarbocyanine iodide was used to detect cell membrane potential. In Figure 5B, the control group was presented with much higher fluorescence intensities than the 2-, 4-, and 8-μg/ml chrysin treatment group and the 4- and 8-μg/ml chrysin combination-treatment group. The cumulative results indicated that the mechanism of the synergistic effect involved the destruction of the inner membrane of bacteria along with a change in the potential of the membrane.

### Synergic effects on a biofilm by CLSM

**Figure 6:**
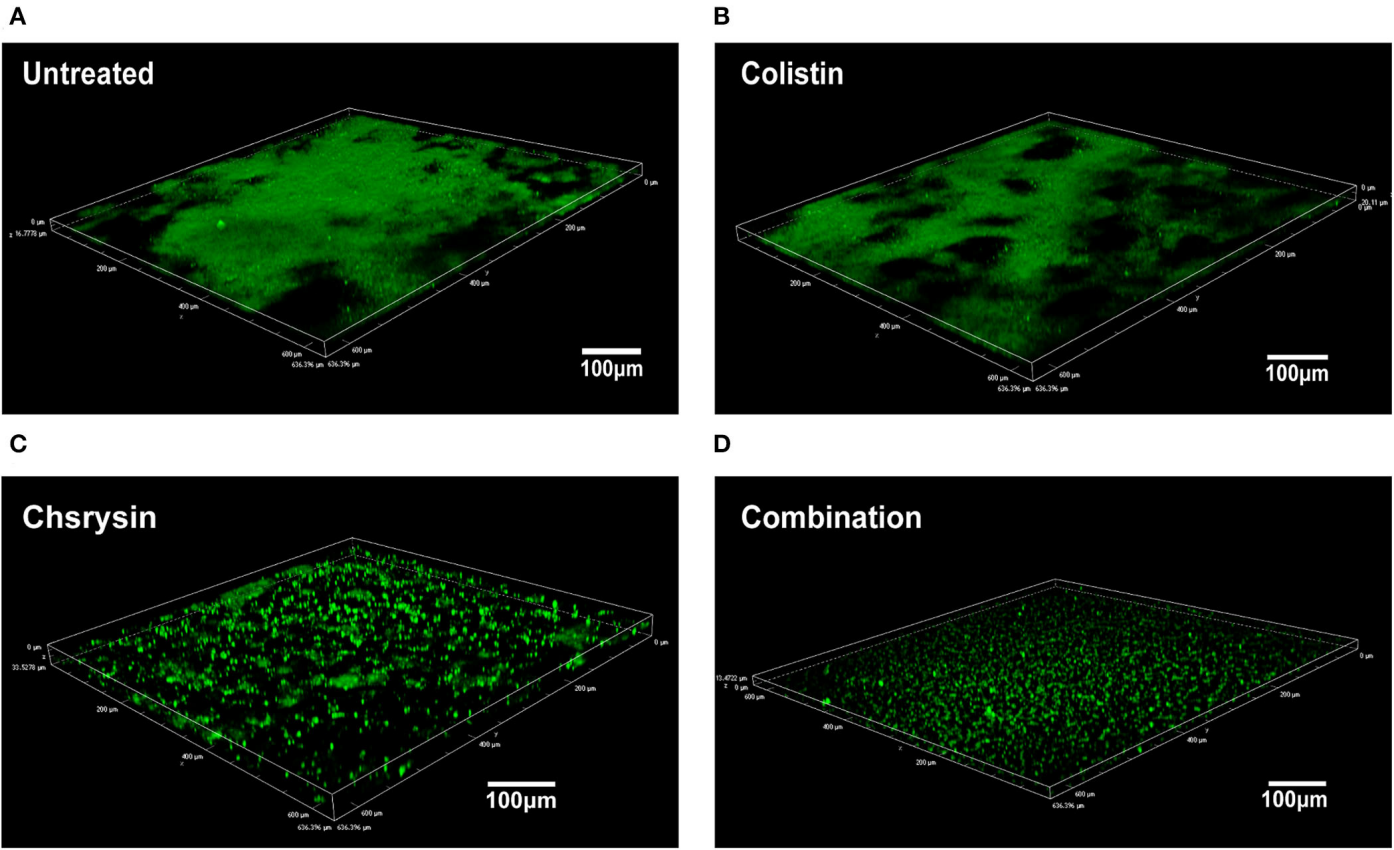
Confocal Laser Scanning Microscopy (CLSM) of the effects of chrysin and colistin combination therapy on bacterial biofilm formation of COL-R *A. baumannii* BM2622. **(A)** Luria Bertani (LB) broth control at **×**20 magnification. **(B)** Colistin alone at **×**20 magnification. **(C)** Chrysin alone at **×**20 magnification. **(D)** Chrysin/colistin combination at **×**20 magnification.

To further explore the effect of chrysin/colistin combination on a biofilm, the microscopic examination was performed by CLSM. The images revealed that untreated *A. baumannii* cells exhibited a thick biofilm (Figure 6A). As shown in Figure 6B, the group treated with 0.25 μg/ml of colistin also produced lot of biofilms. However, 8-μg/ml chrysin-treated group exhibited a dispersed biofilm, and samples treated with the combination of chrysin and colistin exhibited a significant reduction in biofilm biomass (Figures 6C,D).

### The expression of biofilm-associated genes

**Figure 7:**
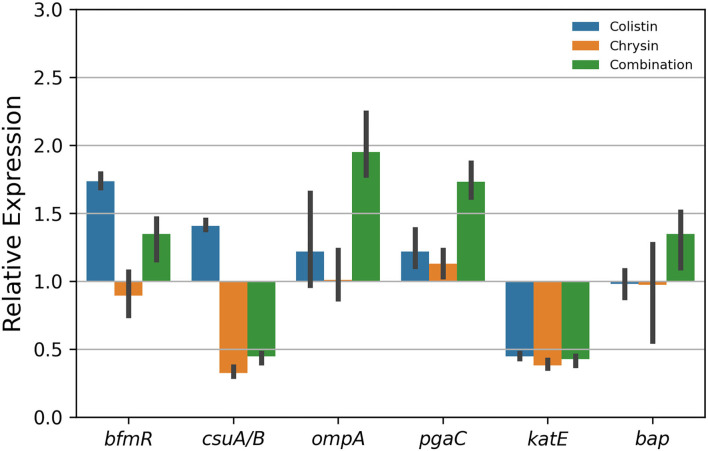
Gene expression of the biofilm of *A. baumannii* BM2622.

To further investigate biofilm formation relative to gene expression after treatment with the combination of colistin and chrysin, quantitative real-time PCR was performed. The results revealed a decrease in the expression of *csuA/B* and *katE* (Figure 7). The expression of *csuA/B* may be attributed to chrysin, owing to the relative expression of this gene in the chrysin-only treatment group, which is < 0.5. Colistin or chrysin was found to decrease the expression of *katE*, therefore; the expression was decreased in the combination group. No difference was noted in the expression of the other four biofilm-related genes among various treatment groups. In summary, this combination exhibited different effects on the biofilm-related gene.

## Discussion

*Acinetobacter baumannii* belongs to *Acinetobacter* spp, which has been regarded as an opportunistic pathogen that is associated with several nosocomial infections such as pneumonia, bloodstream infections, and cephalomeningitis. Infectious diseases caused by *A. baumannii* pose a threat to the global healthcare system (Mea et al., 2021). The strain characteristics of *A. baumannii* are prone to acquire drug resistance. Moreover, genomic sequence analysis of several MDR isolates revealed the presence of several large genomic islands (e.g., AbaR1, R2, R3, and R5) containing multiple resistance genes that were believed to have been acquired from other Gram-negative bacterial genes (Gordon and Wareham, 2010), which contributed to the ineffectiveness of antibiotics, even for colistin. Interestingly, several non-traditional drugs have been shown to recover the sensitivity of MDR bacteria (Ayerbe-Algaba et al., 2018; Pollini et al., 2018; Nguyen et al., 2021).

Several plant extract materials, which include flavonoids with different biological activities that have attracted significant attention, have been extensively studied. Wang et al. (2018) reported that quercetin could significantly eradicate *P. aeruginosa, S. aureus*, and *Escherichia coli*. Another study explored the mechanism of antimicrobial activity and found that flavonoids could induce cell membrane structural damage, which is fatal for bacteria (Tagousop et al., 2018). Similarly, our findings revealed that chrysin could destroy the bacterial outer membrane and alter the cell membrane potential (Figure 5). Furthermore, rutin, morin, and quercetin were reported to function as an effective antibiotic adjuvant against methicillin-resistant *S. aureus* (Amin et al., 2015).

Chrysin is a type of flavonoid that can be extracted from plants or other natural materials such as honey. It is considered a promising phytochemical for broad-spectrum biological activities, such as antidiabetic and anticancer properties, as well as for showing benefits for cardiovascular health (Ciftci et al., 2012; Liu et al., 2016; Kang et al., 2017). Moreover, chrysin has been proven to reduce colistin-mediated side effects, such as renal injury and reproductive damage (Aksu et al., 2018; Hanedan et al., 2018). Our finding indicated that chrysin could strongly enhance the antimicrobial activity of colistin against *A. baumannii*. Therefore, chrysin can be a promising adjuvant for colistin, as it not only reduces antibiotic-induced side effects but also optimizes the clinical drug dosage of colistin.

Biofilm formation is attributable to nosocomial infection and recurrent infections caused by *A. baumannii*, which plays an important role in drug resistance and virulence (Colquhoun and Rather, 2020). Adhesion is the first step involved in the switch of planktonic bacteria to a biofilm phenotype. The chaperone-usher pili (Csu) assembly system, including pilin subunits (CsuA/B, CsuA, CsuB, and CsuE) and transport proteins (CsuC and CsuD), is conserved for *A. baumannii* to attach to the surface of medical appliances (de Breij et al., 2009) and protein A (Omp A) and Biofilm-Associated Protein- (Bap-) mediated adhesion on host cells, such as epithelial cells (Gaddy et al., 2009; Valle et al., 2012). Disruption of the CsuA/BABCDE type I pilus system can lead to a severe decrease in biofilm formation on the non-biological surface (Yoon et al., 2015). As shown in Figure 7, the expression of *csuA/B* was downregulated by chrysin, which might play a role in reducing the amount of biofilm. Some studies reported that a regulator BfmR, which is related to a two-component signal transduction system, is engaged in the interaction of *A. baumannii* and the host immune system (Russo et al., 2016), while the protein poly-N-acetylglucosamine (PNAG) performs a function similar to that by *bfmR*, which is encoded by a pgaABCD operon (Choi et al., 2009). RpoS plays an important role in mature biofilm formation by inducing motility-related genes and by repressing the synthesis of a biofilm exopolysaccharide, and *katE* is a reporter gene of RpoS (Ito et al., 2008; Wang et al., 2011; Lories et al., 2020). Therefore, when the expression of *katE* was downregulated by chrysin and colistin (Figure 7), biofilm formation was also inhibited. As shown in Figure 6, a microscopic examination of CLSM also demonstrated that chrysin disrupts the integrity of the biofilm. Similarly, the combination of chrysin and colistin could reduce bacterial activity and biofilm thickness. These results together support that biofilm biomass decreases as a result of the treatment.

In this study, we detected, for the first time, that chrysin could significantly enhance the sensitivity of *A. baumannii* to colistin, irrespective of the presence or absence of COL-R phenotype. Biofilm formation of *A. baumannii* could be inhibited by the combination of chrysin and colistin, and this combination demonstrated the ability to destroy the membrane and alter the bacterial membrane potential, which were considered to be the mechanisms of synergy. Importantly, chrysin did not demonstrate cytotoxicity on RAW264.7, and the immunocyte showed higher cell viability, implying that chrysin might protect the immune system. Moreover, chrysin and colistin exhibited biological synergy not only *in vitro* but also *in vivo*, implying that the activity of the combination group was unaffected by humoral-mediated immunity, exhibiting a significant potential for clinical application.

## Conclusion

In summary, our study results report the synergistic effect of chrysin in combination with colistin against COL-R and COL-S *A. baumannii* strains. This combination treatment enhanced the bactericidal effect of colistin, whereby chrysin could slow down the development of colistin resistance in clinical strains of *A. baumannii*. The limitations of our study were that we did not explore the interaction of other classes of antibiotics with chrysin and the resulting effect on other Gram-negative bacteria. Research on chrysin as a promising adjuvant to colistin indicated the possibility of flavonoids in the treatment of bacterial infections. Our cumulative findings may confirm the potential efficacy of chrysin to treat clinically isolated *A. baumannii*.

## Data availability statement

The raw data supporting the conclusions of this article will be made available by the authors, upon reasonable request.

## Ethics statement

All animal studies were approved by the Zhejiang Association for Science and Technology SYXK [ID: SYXK (Zhejiang) 2018-0017] and conducted in accordance with Wenzhou Laboratory Animal Welfare and Ethics guidelines.

## Author contributions

YZ conducted the experiments, analyzed the data, and wrote the manuscript. YL, LF, MX, HW, and ZY participated in the experiments. QW and CZ participated in the analysis of the results. TZ and JC helped design the study. All authors contributed to the article and approved the submitted version.

## Funding

This work was supported by the Key Laboratory of Clinical Laboratory Diagnosis and Translational Research of Zhejiang province (Grant No: 2022E10022), the Major Projects of Wenzhou Science and Technology Bureau (ZY2019011), and the Planned Science and Technology Project of Wenzhou (No. Y20170204).

## Conflict of interest

The authors declare that the research was conducted in the absence of any commercial or financial relationships that could be construed as a potential conflict of interest.

## Publisher's note

All claims expressed in this article are solely those of the authors and do not necessarily represent those of their affiliated organizations, or those of the publisher, the editors and the reviewers. Any product that may be evaluated in this article, or claim that may be made by its manufacturer, is not guaranteed or endorsed by the publisher.
