# Supplementary material for: In vitro and in vivo synergistic effect of chrysin in combination with colistin against Acinetobacter baumannii
Source: Front Microbiol. 2022 Oct 28;13:961498. doi: 10.3389/fmicb.2022.961498 (PMC9650306; doi:10.3389/fmicb.2022.961498)
Supplement: Supplementary file 1 [file Table_1.DOCX]

**Supplementary Table 1 |** Primer sequence used in this study.

| **Genes** | **Forward primer** | **Reverse primer** |
| --- | --- | --- |
| *bfmR* | 5'-CTG GTA GGT AAT GCA GTT CG-3' | 5'-GAG AGA CCC AAA CCA TAA CC-3' |
| *csuA/B* | 5'-ATG CGG TAA ATA CTC AAG CA-3' | 5'-TCA CAG AAA TAT TGC CAC CT-3' |
| *ompA* | 5'-CTC TTG CTG GCT TAA ACG TA-3' | 5'-GCA ATT TCT GGC TTG TAT TG-3' |
| *pgaC* | 5'-CAG TGG TAT GGC GTG ATA TT-3' | 5'-GGT ACT GCA ACA ACA CTG GT-3' |
| *katE* | 5'-GTG TCC GGT TCA GGT TTT AC-3' | 5'-GGA TTC TTG ACA GAC CCA AC-3' |
| *rplB* | 5'-GGT CGT AAT AAC AAC GGT CA-3' | 5'-AAT AAT GCA ATA TGC GCT GT-3' |
| *bap* | 5'-CCT TGGT AAC CAC AGA GGG A-3' | 5'-TGA CTG CAT TGG TAC CCT CC-3' |
